# Comparison of ANFIS and ANN modeling for predicting the water absorption behavior of polyurethane treated polyester fabric

**DOI:** 10.1016/j.heliyon.2021.e08000

**Published:** 2021-09-15

**Authors:** Joy Sarkar, Zawad Hasan Prottoy, Md. Tanimul Bari, Md Abdullah Al Faruque

**Affiliations:** a Department of Textile Engineering, Khulna University of Engineering & Technology, Khulna 9203, Bangladesh; b Department of Fabric Engineering, Bangladesh University of Textiles, Dhaka 1208, Bangladesh; c Institute for Frontier Materials, Deakin University, Geelong, Victoria 3216, Australia

**Keywords:** ANFIS, ANN, Modeling, Textile substrates, Functional finishing

## Abstract

Nowadays, the polyurethane and its derivatives are highly applied as a surface modification material onto the textile substrates in different forms to enhance the functional properties of the textile materials. The primary purpose of this study is to develop prediction models to model the absorption property of the textile substrate using the Adaptive Neuro-Fuzzy Inference System (ANFIS) and Artificial Neural Network (ANN) methods. In this study, polyurethane (PU) along with acrylic binder was applied on the dyed polyester knitted fabric to develop and validate the prediction models. Through the morphological study, it was evident that the solution prepared with the polyurethane and the acrylic binder was effectively coated onto the fabric surface. The ANFIS model was constructed by considering binder (ml) and PU (%) as input parameters, whereas absorbency (%) was the only output parameter. On the other hand, the system was trained with 70% data for constructing the ANN model whereas testing and validation were done with 15% data, respectively. To train the network, feed-forward backpropagation with Levenberg–Marquardt learning algorithm was used. The coefficient of determination (R^2^) was found to be 0.98 and 0.93 for ANFIS and ANN model, respectively. Both prediction models exhibited an excellent mean absolute error percentage (0.76% for the ANFIS model and 1.18% for the ANN model). Furthermore, an outstanding root-mean-square error (RMSE) of 0.61% and 1.28% for ANFIS and ANN models was observed. These results suggested an excellent performance of the developed models to predict the absorption property of the polyurethane and acrylic binder treated fabric. Besides, these models can be taken as a basis to develop prediction models for specific types of functional applications of the textile materials to eliminate heaps of trial and error efforts of the textile industries, which eventually be helpful in the scalable production of functional textiles.

## 1 Introduction

To enhance the functional properties of the textile fabrics, a number of approaches such as coating is used enormously [1, 2, 3]. Textile fabrics manufactured with both the natural and synthetic fibres, having different forms, size, and shape have been treated with various kinds of materials to impart the functional properties for diversified applications [4, 5]. Polyurethane and its derivatives are also being used extensively to enhance several properties of the textiles, especially the water repellency property [6, 7]. Generally, the water repellency indicates that the material is either hydrophobic or repels water upon contact with the textile surface [8]. Water repellency has a close but inverse effect on the water absorption. The textile substrate which absorbs more water is less water repellent, as a rule of thumb. As the water repellency is commonly measured visually by comparing with the references, the objective measurement of water absorption can significantly shed light numerically on the water repellency property of the substrate [9].

Polyester is generally a hydrophobic material that doesn't necessarily confirm that it is water repellent [10]. However, to use in products where water repellency is a must, the polyester also needed to be treated with functional materials [11, 12]. Moreover, as polyester is cheap, it can be used to satisfy water repellency and/or waterproofing applications such as raincoats or umbrellas. From the literature, it has been found that the mechanical properties of PU-coated knitted fabrics have been studied and the results were compared with the regression models [13]. The authors concluded that both the fabric thickness and coating thickness possessed a major impact on the PU-coated knitted fabrics [13]. In other studies, the finishing of textile materials in the forms of membrane, web, and fabrics with PU have also been studied. The researchers were tried to improve the breathability of the treated textiles and the improvement of their hydrophobicity. From these studies, it was prominent that the PU can incorporate the water repellent property of the treated materials and can assist in improving the breathability [14, 15]. Furthermore, the application of PU as flame retardant finish, anti-ultraviolet ray finish, chemical protection finish has also been studied. It has been found that PU with suitable substrate material and in proper form can improve these properties in a notable manner [16, 17, 18]. However, as per our best knowledge, no study has been reported to improve the water repellent property of the polyester knit fabrics by treating with PU and acrylic binder. The use of PU and binder with polyester fabrics can open a new horizon of developing a cheap, functional finishing process of the textile fabrics that can assist in the large-scale production of the functional textiles and can be used in diversified application areas. As the final property plays a significant role for any functional finished textiles, the property prediction of the mentioned type of fabrics can also support the scalable production of cheaper functional fabrics, especially in the developing countries like Bangladesh, where inexpensive but functional fabrics can be a prevalent choice. Therefore, we have treated the polyester knit fabric with polyurethane (PU) and acrylic binder in this study. Furthermore, various models' property prediction of the PU-treated materials can be an excellent opportunity to understand the effect of finishing and quality of the final product.

Among most of the common and available models such as mathematical, statistical, and soft computing-based models for predicting the properties of the textile substrate, the soft computing-based methods are found to be more accurate and suitable comparatively because of their exhibition of capability to address the nonlinear relationship of the process parameters and output parameters of the textiles [19, 20, 21, 22, 23]. Whereas, the models like mathematical and statistical produce noisy data with a lower degree of accuracy [24, 25, 26]. As soft computing-based methods like ANFIS or ANN can substantially address the issue with reasonable accuracy, soft computing approaches can be used in this regard [27]. Moreover, these methods have been extensively used in other prominent fields like engineering, agriculture, and medical to significantly model and predict the data [28, 29, 30]. An extensive literature review revealed that soft computing-based approaches have widely been used in the textile industry to model and predict the properties of different materials with admirable accuracy. Among different soft computing-based approaches, the fuzzy inference-based prediction system has been successfully developed and utilized in the prediction of different fabric properties [25, 31, 32, 33, 34]. Besides, the color property [35, 36], tearing strength, and seam strength of the garments [37, 38] have also been predicted using the fuzzy inference-based prediction system. Although the fuzzy logic-based models exhibited excellent performance in most cases, it primarily relies on expert suggested rule base system, which can be subjective depending upon the expert. On the other hand, the ANFIS and ANN based models can operate on the principle of the artificial network that can eliminate the subjective error of the rule base of the fuzzy inference system. In textiles, ANFIS and ANN are also extensively used in property prediction [20, 39, 40, 41, 42, 43]. However, as per the authors' best knowledge, no significant work has been reported to predict the water absorption of a treated textile substrate by ANFIS and/or ANN models.

Commonly, the ANN and ANFIS model shows better performance with a larger amount of data that is labor-intensive to obtain and may not always be possible in an industrial situation [44, 45]. But it is not uncommon to use a limited amount of data to successfully design models and predict different properties with satisfactory accuracy [24, 39, 46]. Hence it is clear that laboratory-scale experimental data can also be used to train the ANN and ANFIS models to predict data with distinctive accuracy. Therefore, in this study, both the ANFIS and ANN methods have been developed to predict the water absorbency of the PU-treated 100% polyester knitted fabric. The developed models have also been validated using the trial data. These models can act as principles to the development of other models for artificial intelligence-based prediction systems for the treated textile materials provided that the models perform satisfactorily.

## 2 Materials and methods

### 2.1 Development of ANN and ANFIS prediction model

#### 2.1.1 The basic structure of artificial neural network (ANN)

The long trail of adaption and development has provided many attractive features to the human brain like extensive parallelism, distributed representation and computation, learning ability, capacity for generalization, adaptability, processing of inherent contextual information, fault tolerance, and low energy consumption. Artificial neural networks (ANNs) are highly parallel computing systems inspired by biological neural networks [47]. ANNs were first established in the 1950s to emulate the architecture of the biological brain of humankind [48]. The ANN can develop an internal representation of a signal pattern introduced to the network as an input. This automated processing or "learning" is achieved by dynamically changing the strengths of network interconnection (adaptive weights) associated with each neuron [49]. A neural network contains a vast number of interacting neurons, like that of the human brain. However, an artificial neural layout is more straightforward than a biological one [50].

**Figure 1:**
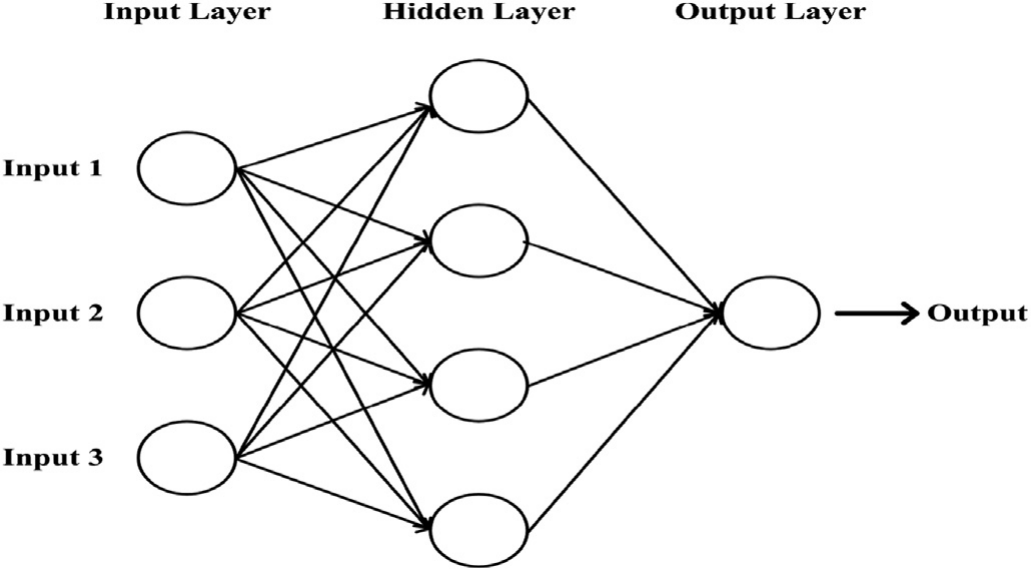
The general architecture of a neural network.

An artificial neural network (or merely a neural network) consists of input neuron layers (or nodes, units), one or more hidden neuron layers, and a final layer which consists of the output neurons. In Figure 1, the general architecture of an ANN has been illustrated. A numeric value called weight is aligned with each connection. The output, $h_{i}$ from the final layer of neuron iin the hidden layer can be expressed as Eq. (1) [51].

(1) $$h_{i}=\sigma \left(\overset{N}{\underset{j=1}{\sum }}V_{ij}x_{j}+T_{i}^{hid}\right)$$

Where, σ = Activation function, N= Number of input neurons, $V_{ij}$ = Weights, $x_{j}$ = Inputs of the input neurons, and $T_{i}^{hid}$= Threshold terms of the hidden neurons.

To integrate the nonlinearity into the neural network, the objective of the activation mechanism is to connect the value of the neuron so that the divergent neurons do not paralyze the neural network.

ANN uses a learning method to predict the output of a given input. Learning of ANNs can be classified into two major categories: supervised learning and unsupervised learning. In supervised learning, training is needed to assist the system in predicting the output. Weights are adjusted to desired values in the training to minimize the errors. In those training, examples of previous data are provided in which inputs and corresponding outputs are given to the ANN system. Some further special considerations are needed to minimize the error of the result. On the contrary, the unsupervised training does not provide any previous example in its database, and ANN tries to figure out the output through the patterns and trends [52].

Artificial Neural Networks (ANN) possess several merits, such as ANN emulating the human brain so that it can do operations while learning. In addition, ANN can perform its organization while carrying out tasks, which is not possible for regular computer programs. Besides, ANN can work parallelly, which is not possible for ordinary computer programs. Moreover, ANN is reasonably fast while human brain processing is much slower than ANN [53]. Although ANN has a tremendous amount of advantages, it contains some limitations too. For example, there is no established method of operation for ANN. Very often, the quality of the final output can be unpredictable and erroneous. Furthermore, most ANN programs do not provide a solution and insight into fixing problems discovered from the final output [54]. Another major issue with ANN is overfitting: in the output, ANN provides a larger value of error than the smaller error that is provided in its training set [55]. Despite these disadvantages, ANN is frequently used to solve many scientific problems in present days for its advantageous counterparts.

#### 2.1.2 Development of ANN model

In this research, we have used NN toolbox of MATLAB (Version 9.6). In the feed-forward neural network, input variables were PU (5–20 %), and binder (2–10 ml). The absorbency (%) was chosen as the output variable in the output layer. The model was constructed by using a 2-4-1 structure, which means the network was developed by using 2 neurons for the input layer, 4 neurons for the hidden layer, and 1 neuron for the output layer. No transfer function was used in the input layer, whereas the log-sig transfer function in the hidden layer and purelin transfer function in the output layer has been used. A feed-forward backpropagation with Levenberg–Marquardt learning algorithm was employed to train the network. A total of 20 datasets were used for constructing the ANN prediction model. Among the datasets, 70% (14 datasets) were used to train the system whereas the rest 30% datasets were equally distributed for testing and validation purpose. It is not uncommon for smaller datasets to use one test-set for both validation and testing [24]. Therefore, in this study, all 30% (6 datasets) were used as the test-set to compare the results with the experimental and ANFIS model predicted results. Moreover, the datasets for testing the model were selected randomly to test the ANN prediction model.

#### 2.1.3 The basic structure of adaptive neuro-fuzzy inference system (ANFIS)

It is unlikely that a model based on arbitrarily established and unpredictable processes will work out with traditional mathematical tools (e.g., differential equations). On the other hand, a fuzzy inference method that uses fuzzy if-then rules has a good probability to model the qualitative dimensions of human understanding and reasoning even without accurate quantitative analysis [56, 57]. One of the most effective artificial intelligence approaches is fuzzy logic, developed by Zadeh [58]. We must face different circumstances in everyday life involving uncertainty. The fuzzy inference method enables the use of the decision-making process to express ambiguous circumstances in the form of rules. It has therefore been used to resolve numerous problems [59, 60]. Being the association of ANN and fuzzy networks, neuro-fuzzy systems typically have the benefit of making things more straightforward than before when conventional neural networks were being used [61].

**Figure 2:**
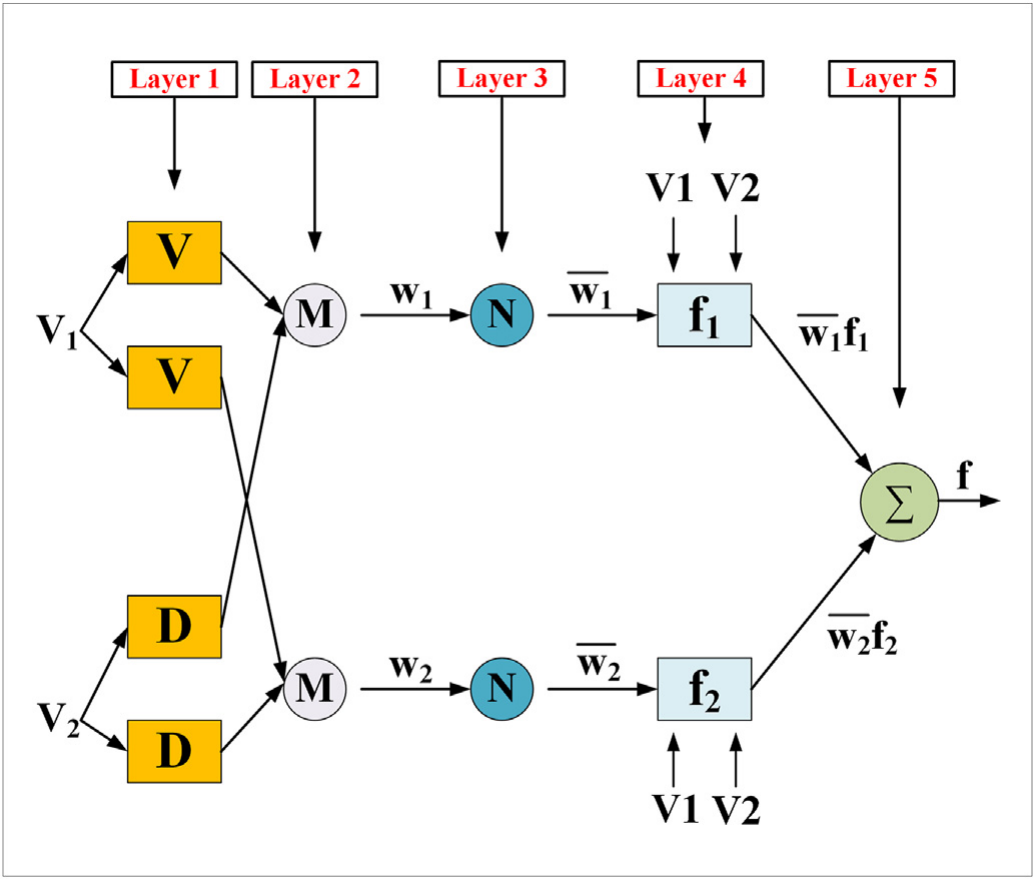
General architecture of ANFIS structure.

There are five layers in the architecture of ANFIS, namely fuzzy layer, product layer, normalized layer, de-fuzzy layer, and total output layer. All those 5 layers are shown in Figure 2.

For convenience, the fuzzy inference method can be considered consisting of two inputs v and d, and one output f. A succinct overview of the five layers of the ANFIS algorithm is given below [61].

Layer 1 is a fuzzy layer in which each node is an adaptive one. In this layer, v and d are the input of the system and $O_{1}$ is the output of layer 1's $i^{th}$ node. All the adaptive nodes are square nodes with square functions, which can be represented as Eqs. (2) and (3).

(2) $$O_{1,i}=\mu_{v}{,}_{i}\left(V\right)for\;i=1,\;2$$

(3) $$O_{1,\;j}=\mu_{d,\;j}\left(V\right)for\;j=1,\;2$$

In this equation, output functions are shown by $O_{1}{,}_{i}$ and $O_{1}{,}_{j}$ and membership functions are shown by $\mu_{v}{,}_{i}$ and $\mu_{v}{,}_{j}$. If we choose a triangular function,

(4) $$\mu_{vi}\left(V\right)=max\left[\min\left(\frac{v-a_{i}}{b_{i}-a_{i}},\;\frac{c_{i}-v}{c_{i}-b_{i}}\right),\;0\right]$$

$\left\{a_{i},b_{i},c_{i}\right\}$ are parameters of triangular membership functions. Again, if we want $\mu_{vi}\left(V\right)$ to be bell-shaped,

(5) $$\mu_{vi}\left(V\right)=\frac{1}{1+\left\{{\left(\frac{v-c_{i}}{a_{i}}\right)}^{2}\right\}b_{i}}$$

Layer 2 gets the input value $v_{i}$ from the first layer and it explores the weights of each membership function. Nodes of this layer are fixed and labeled with M and the product of all arriving signals is used to calculate the output. The output of this layer can be represented in Eq. (6).

(6) $$O_{2,\;i}=w_{i}=\mu_{v,\;i}\left(V\right).\mu_{Dj}\left(d\right),\;i=1,\;2$$

Nodes in layer 3 are labeled with N, which suggests normalization to the firing strength from the previous layer. This layer conducts Pre-condition matching of fuzzy rules. The output of this layer is represented as $\bar{w_{i}}$ which is

(7) $$O_{3,\;i}=\bar{w_{i}}=\frac{w_{i}}{w_{1}+w_{2}}$$

Output values provided by layer 4 result from the inference of rules. The output is a first-order polynomial and product of normalized firing rule strength. Weighted output represented by node function:

(8) $$O_{4,i}=\bar{w_{i}}f_{i}=\bar{w_{i}}\left(p_{i}v+q_{i}d+r_{i}\right),\;i=1,\;2$$

$p_{i}.q_{i},\;and\;r_{j}$ are called linear or consequent parameters and $O_{4,i}$ is the output.

Layer 5 is the output layer which sums up all the incoming values from layer 4 and transmutes all fuzzy classification results into solid values. The summation of all the input signals is conducted by Eq. (9).

(9) $$O_{5,i}=\underset{i}{\sum }\bar{w_{i}}f_{i}=\frac{\sum_{i}w_{i}f_{i}}{w_{1}+w_{2}},\;i=1,\;2$$

During learning the information of a dataset, ANFIS computes the membership function parameters, which change throughout the learning process to track the input/output data. ANFIS tunes all the parameters that can be manipulated for handling real-world situations. For improving the convergence, the hybrid network can be trained by a hybrid algorithm [61, 62]. A hybrid learning algorithm consists of a forward pass and a backward pass. In the forward pass, node outputs keep moving forward up to layer 4, and the least square method assists the system in identifying the consequent. While in the backward pass, error signals are transmitted backward, and gradient descent updates the premise parameters [63].

The main advantage of the neuro-fuzzy system is, it combines neural network properties with fuzzy logic and hence eliminates the limitation of both. While fuzzy logic deals with the explicit knowledge that can be obtained and understood, neural network deals with implicit knowledge obtained by learning [64]. ANFIS puts fuzzy logic's qualitative approach and neural network's adaptive capabilities into one system [65]. Apart from its advantages, it has some limitations too. In a fuzzy system, membership parameters and rules are established by a trial-and-error process. An intricate system requires a sizeable time to perceive the appropriate membership function and rules to get a well-grounded solution. Also, the generalization potentiality of the fuzzy system is very poor [64].

#### 2.1.4 Development of ANFIS model

For the ANFIS modeling, the fuzzy toolbox of MATLAB (version 9.6) was used for modeling the data. Binder (ml) and PU (%) were taken as the input parameters whereas the absorption (%) was the only output parameter. 100 training epochs were selected to train the ANFIS model. The trimf type membership function (MF) was chosen for the input side, whereas, for output, the linear type of membership function (MF) was selected. Three linguistic variables for the input parameters as Low (L), Medium (M), and High (H) were used. Among 20 datasets, 70 % (14 datasets) were used for training the model, whereas the remaining 30% (6 datasets) were used to test the model. The datasets to test the model were selected randomly from the overall datasets.

### 2.2 Experimental procedure and data acquisition

#### 2.2.1 Materials

100% dyed polyester single jersey fabric of 160 g per square meter (GSM) was used. The fabric was Optical Brightening Agent (OBA) treated and white dyed. The fabric was provided by a textile manufacturing factory located at Gazipur, Dhaka, Bangladesh. Polyurethane and acrylic binder were purchased from the City Scientific Store, Khulna, Bangladesh.

#### 2.2.2 Materials preparation

The collected fabric was treated with polyurethane along with an acrylic binder. The polyester fabric was soaked into the solution made by using a specific amount of distilled water, PU, and acrylic binder. The Polyurethane solutions were prepared in a concentration of 5%, 10%,15%, and 20%. The concentration of the acrylic binder was chosen as 2 ml, 4 ml, 6 ml, 8 ml, and 10 ml. For 5% PU, 5 ml PU was added to 95 ml water. Similar approaches were followed for each percentage of the PU. With the total 100 ml PU and water solution, a specific amount of acrylic binder was used. The final solutions were prepared with the help of a magnetic stirrer (MTOPS, Korea). The stirring speed was maintained at 1000 rpm. The samples were then impregnated into their respective solution for 15 min for complete impregnation of the solution to the polyester fabric followed by the squeezing in a laboratory padding mangle (GESTER, China) for the removal of residual polyurethane solution from the fabric. The diameter of the roller of the padder was 125 mm, and the roller hardness was the 70-degree shore. The pressure used in the padding was 0.4 kg/cm^2,^ and the cloth speed was kept at 3 m/min to obtain a take-up percentage of 70 %. The treated samples were then dried in the oven (GESTER, China) at 100 °C for 10 min. The treatment condition which was used for testing the developed models is presented in Table 1.

**Table 1:** Treatment condition of the samples used for the prediction models.

Sample	PU (%)	Binder (ml)
A	15	02
B	10	04
C	20	06
D	15	08
E	20	08
F	05	10

#### 2.2.3 Evaluation of water absorption

The treated samples were conditioned on a flat surface for 24 h before testing under standard atmospheric conditions at relative humidity (RH) of 65% and temperature of 20 °C [66]. Then the samples were tested for water absorption by following the static immersion method to evaluate water absorption amount according to BS 3449 [67]. The water absorption was calculated by following Eq. (10). An average of five samples was taken as the final reading.

(10) $$Water\;absorption=\frac{Absorbed\;water\;mass\;\left(gm\right)}{Dry\;mass\;\left(gm\right)}$$

#### 2.2.4 Scanning electron microscopy (SEM)

The Phenom Pro Desktop SEM was used to examine the surface morphology of the treated samples. The accelerating voltage was kept at 5 kV.

#### 2.2.5 Statistical analysis

By following the global prediction errors, the performance of the developed ANFIS and ANN models was determined and compared. The prediction errors considered in the study are root-mean-square error (RMSE), mean absolute error percentage (MAEP), and coefficient of determination (R^2^). The formulations of the prediction errors are as following:

(11) $$RMSE=\sqrt{\frac{\sum_{i=1}^{i=N}{\left(E_{a}-E_{p}\right)}^{2}}{N}}$$

(12) $$\text{MAEP}=\frac{1}{\text{N}}\overset{i=N}{\underset{i=1}{\sum }}\left(\frac{\left|E_{a}-E_{p}\right|}{E_{a}}\times 100\right)$$

(13) $$R^{2}=1-\left(\frac{\sum_{i=1}^{i=N}{\left(E_{a}-E_{p}\right)}^{2}}{\sum_{I=1}^{I=N}{\left(E_{a}-E_{m}\right)}^{2}}\right)$$

Here, $E_{a}=\text{Actual ​value},\;E_{p}=\text{Model ​predicted ​value},\;E_{m}=\text{Mean ​value},\;\text{and}\;N=\text{Number ​of ​the ​pattern}$.

GraphPad Prism (version 8) was used to analyze the correlation between predicted and experimental results. The same software has been used for the comparative analysis among the experimental and model-predicted values as well.

## 3 Results and discussion

### 3.1 Experimental results

**Figure 3:**
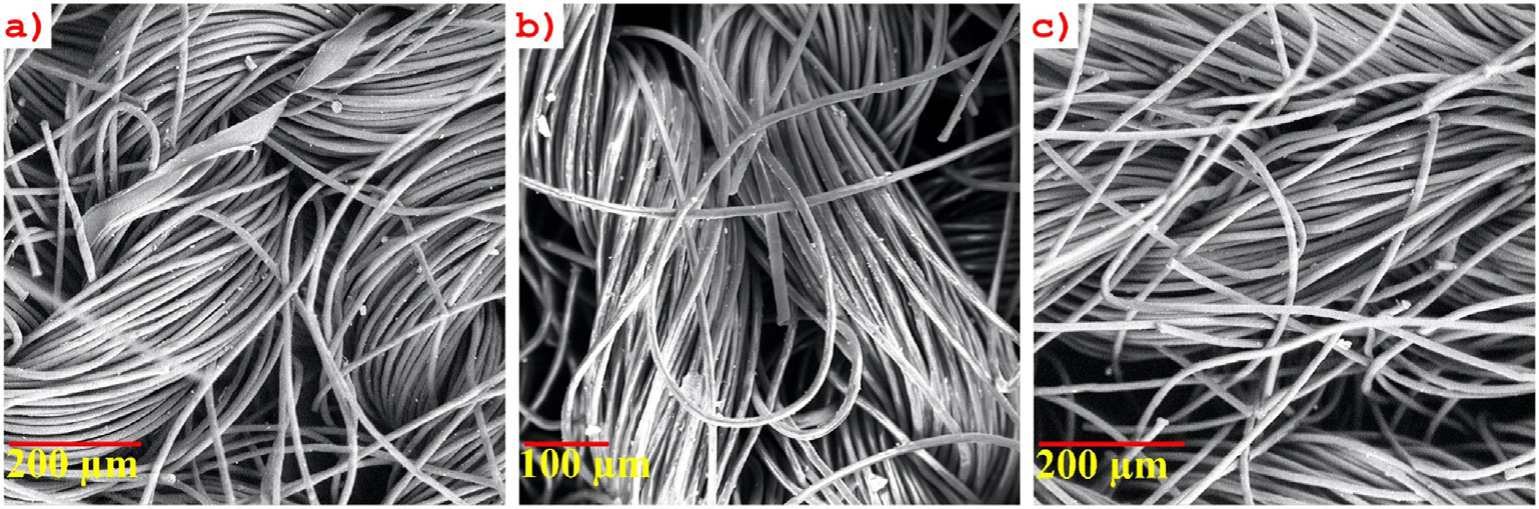
SEM images of the sample. a) Untreated sample, b) 5% PU and 2 ml binder, and c) 20% PU and 4 ml binder.

It was found from the experiment that the application of PU decreased the absorbency of the fabric. The amount of PU and binder both hinder the penetration of water molecules inside the fabric structure. As binders help more PU to bind with the fabric surface, the absorbency decreases. In Figure 3, the scanning electron microscopy shows the treated and untreated fabrics' surface anatomy. From the figure, it can be seen that the PU accumulates on the surface and affects the absorbency of the treated fabric. It was observed that with the increment of PU %, the water absorbency of the treated fabric decreases by nearly 23.4 %. On the other hand, the same trend was exhibited by the amount of binder. The higher amount of binder results in lower water absorbency. The highest value of water absorbency (∼78.6%) was experienced in the lowest concentration of PU (5%) and binder (2 ml) whereas, the lowest (∼60.2%) water absorbency was found for the highest concentration of PU (20%) and binder (10 ml).

### 3.2 Data prediction by ANFIS model

**Figure 4:**
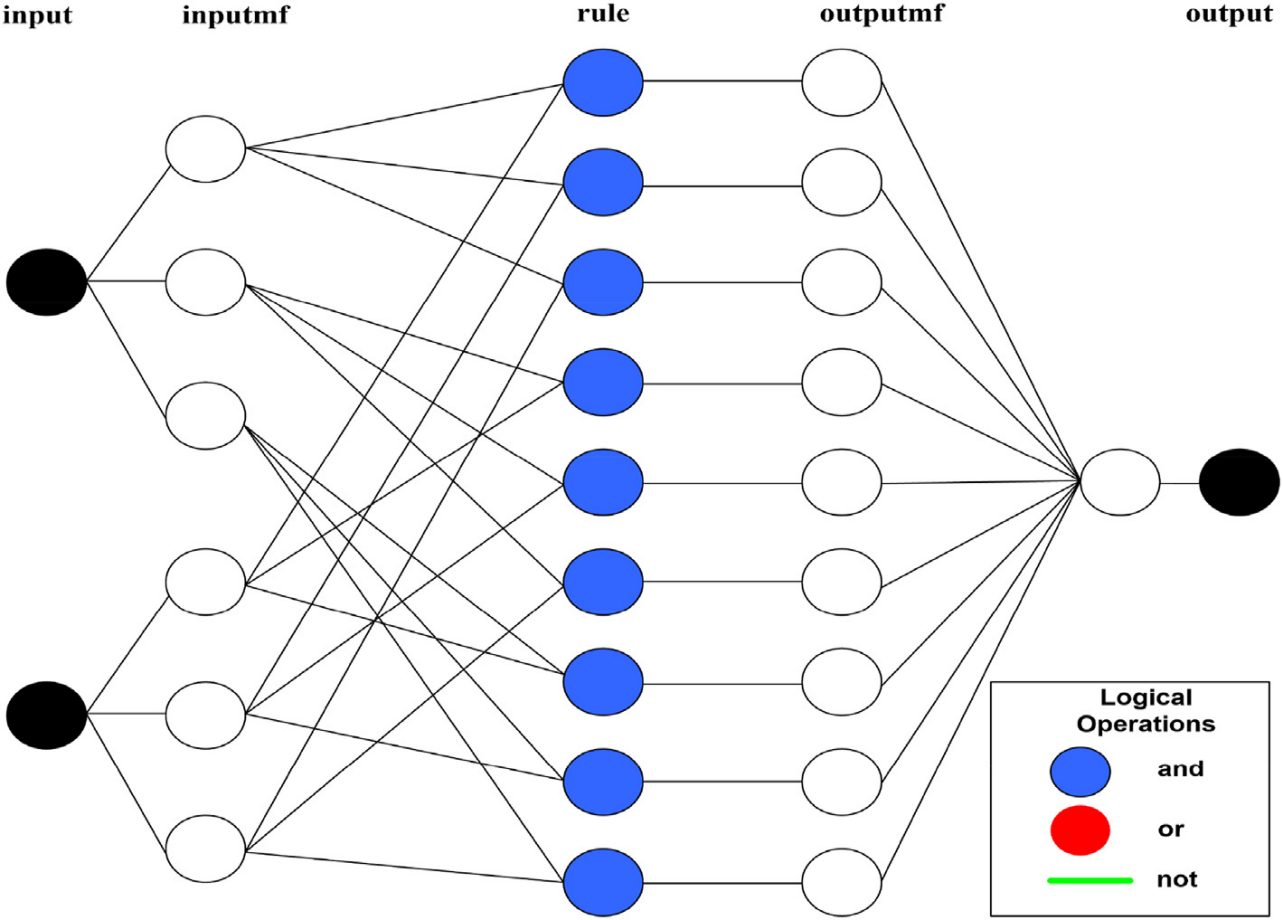
ANFIS model structure.

**Figure 5:**
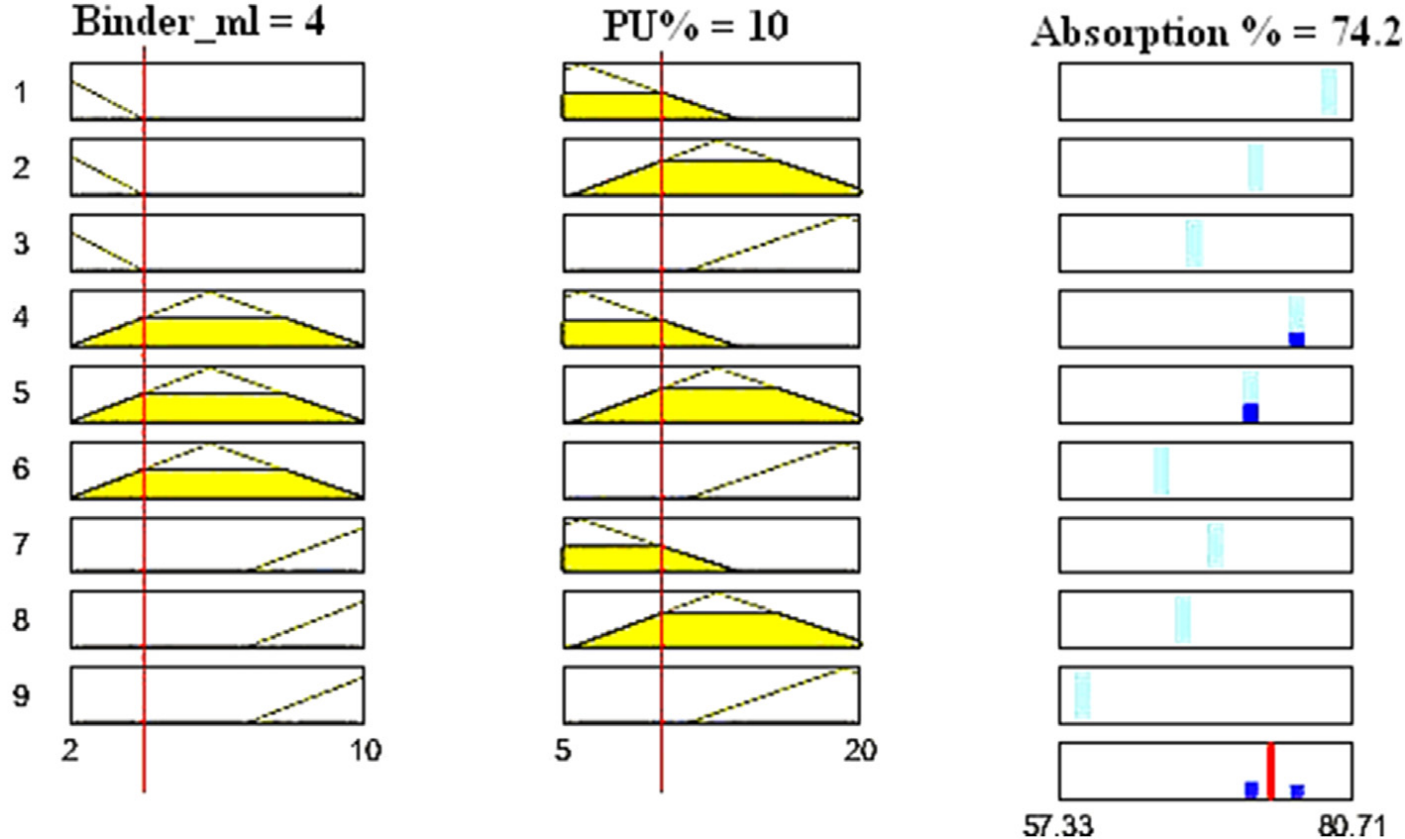
Rule viewer of the ANFIS prediction model.

The basic structure of the ANFIS model for this study is demonstrated in Figure 4. For two input parameters consisting of 03 membership functions (mfs), the system develops 9 'and' based rule bases. Then through the same number of the output mfs, they are converted into a crisp output. On the other hand, the data prediction capability of the ANFIS model has been demonstrated by the rule viewer as presented in Figure 5. For instance, for 10% PU and 4 ml binder, the absorption is ∼74.2 %. The model can predict every output data for every input parameter within the data range. At the same time, for a particular required output, the inputs can be selected accordingly with the rule viewer. As a result, the model can predict output data (absorption %) in response to the input variables (PU % and binder (ml)) and vice versa. The model can be adjusted for a slight change in either parameter to predict the other parameter.

### 3.3 Data prediction by ANN model

**Figure 6:**
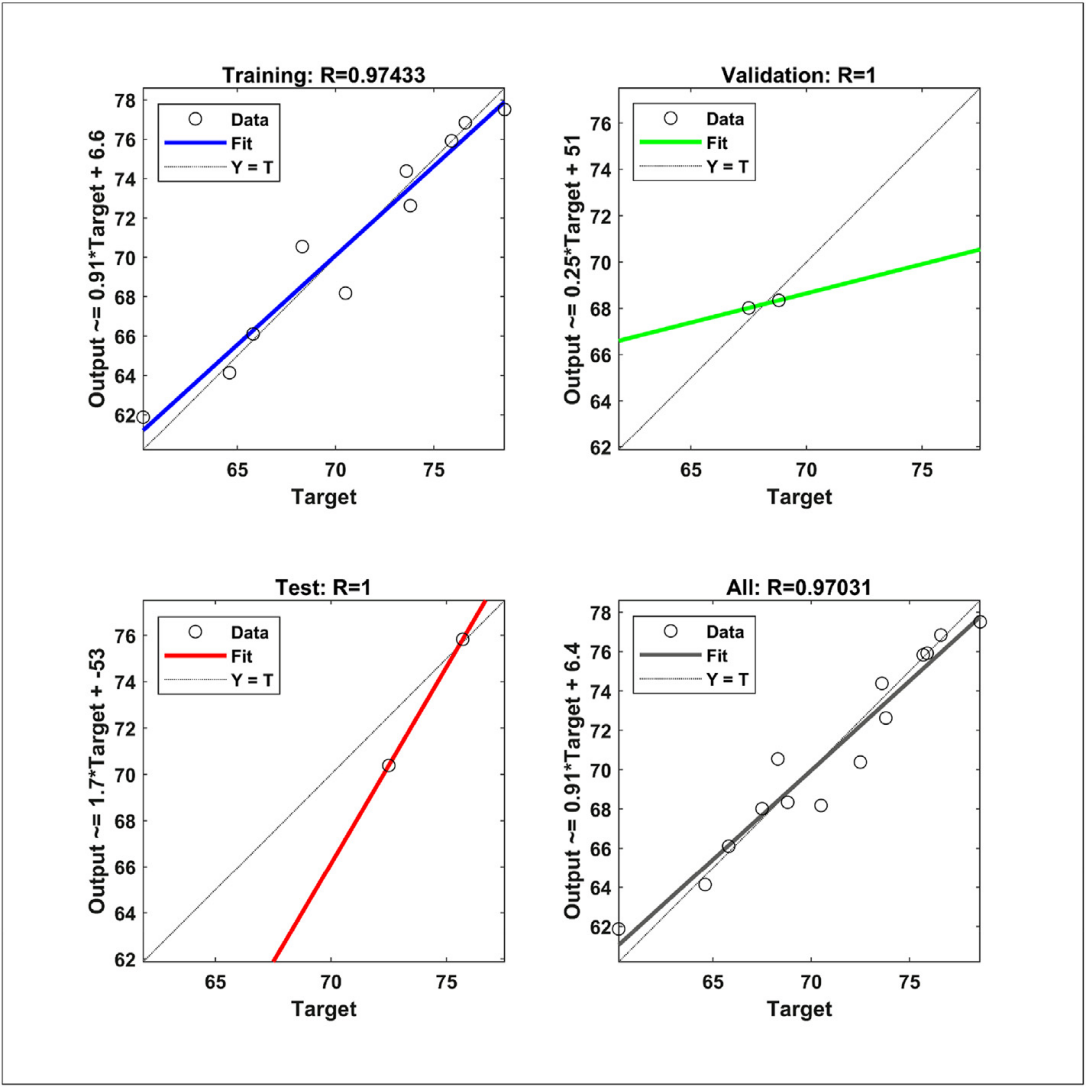
Neural network training regression.

The neural network regression shown in Figure 6 demonstrates the interaction of the network with the raining, testing, and validation data. The correlation coefficient was found 0.974, 1, and 1 for training, testing, and validation data, respectively. Moreover, the straight line presents the linear relationship between the model predicted (output) and experimental (target) data. The results suggest that the actual data are well aligned with the model-predicted data. Hence the model is suitable enough to predict the data with excellent accuracy. The overall correlation coefficient (0.97) confirms the outstanding prediction performance of the developed ANN model.

### 3.4 Comparison between actual and model-predicted results

**Figure 7:**
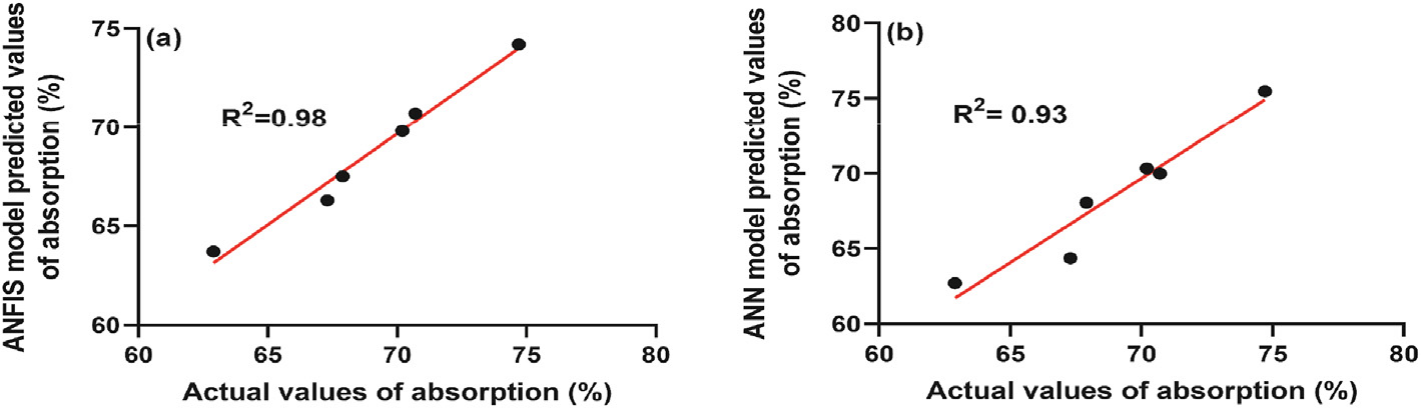
Correlation between actual and (a) ANFIS model predicted absorption (%), (b) ANN model predicted absorption (%).

The comparison and statistical analysis of the actual (experimental) values and the model predicted values of water absorption (%) of the PU-treated polyester fabric are presented in Table 2. It was found that both models have sufficient capability to predict the properties of the treated fabrics. As specific, the mean root-mean-square error (RMSE), mean absolute error percentage (MAEP), and the coefficient of determination (R^2^) was found 0.61, 0.76, and 0.98 respectively for the ANFIS model whereas RMSE, MAEP, and R^2^ values were found as 1.28, 1.18, and 0.93 for the ANN model. As the statistical data for both models fit within the acceptable limit hence proves the suitability of the model to be used in practice. Figure 7 shows the linear fit of the actual and predicted results by a) ANFIS and b) ANN models. The linear fit also suggests the outstanding performance of both models. Though both models are suitable for predicting the water absorption (%) of PU-treated polyester fabric, the ANFIS model performed slightly better in terms of RMSE, MAEP, and R^2^ values.

**Table 2:** Comparison of actual and predicted values of ANFIS and ANN models.

Sample	PU (%)	Binder (ml)	Actual absorption (%)	ANFIS model predicted absorption (%)	ANN model predicted absorption (%)	Absolute error (%) [ANFIS]	Absolute error (%) [ANN]
A	15	02	70.70	70.70	69.98	0.00	1.01
B	10	04	74.70	74.20	75.47	0.67	1.03
C	20	06	67.30	66.30	64.36	1.49	4.37
D	15	08	67.90	67.50	68.04	0.59	0.21
E	20	08	62.90	63.70	62.71	1.27	0.30
F	05	10	70.20	69.80	70.32	0.57	0.16
**Root-mean**-**square error (RMSE)**						**0.61**	**1.28**
**Mean absolute error percentage (MAEP)**						**0.76**	**1.18**
**Co-efficient of determination (R**^**2**^**)**						**0.98**	**0.93**

**Figure 8:**
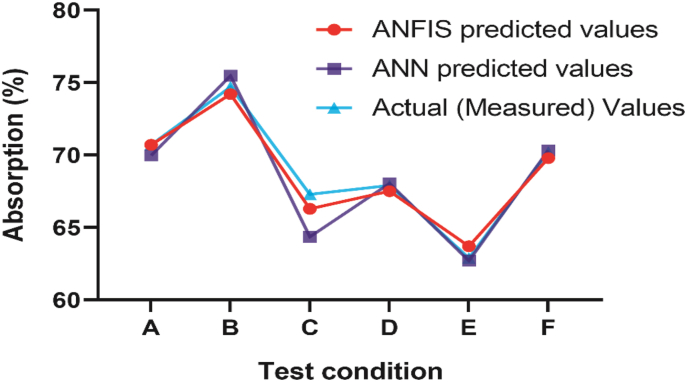
Comparison of ANFIS and ANN predicted results with the actual result for absorption (%).

On the other hand, the original characteristics of the treated fabrics found by the experiment are sustained in the case of the model prediction (for both ANFIS and ANN). As presented in Figure 8, the trend of the behavior of the samples also fits excellently. Table 2 and Figure 8 show that the ANFIS model performed outstandingly for sample A, whereas the ANN model was excellent for sample F, where the absolute error was 0% and 0.16%, respectively. In the case of sample C, both models exhibited a poor performance with slightly higher absolute error (1.49% for ANFIS and 4.37% for ANN), but never exceeded the tolerable limit.

From the prediction errors and Figures 7 and 8, it is certain that the model predicted values are close enough with the experimental values in almost each test condition, along with remarkable similarity in the trend of the behavior of the samples. Therefore, it is evident that the models are suitable with excellent accuracy.

## 4 Conclusion

The findings of the research not only satisfied its primary objectives but also opens a new possibility for predicting the properties of PU-treated textiles. The developed ANFIS and ANN models and their comparison have established the suitability of the models to be used in the practical field. From the analyses, the conclusion can be drawn as:

a) The coefficient of determination (R^2^) was found to be 0.98 and 0.93 for ANFIS and ANN models, respectively. The results imply a good fit between the model-predicted data and experimental data in both cases, indicating the models' suitability and compatibility.
b) The root-mean-square error (RMSE) between the predicted and experimental values of absorption % was found to be 0.61 for the ANFIS model and 1.28 for the ANN model.
c) The mean absolute error percentage (MAEP) between the predicted values and experimental values of absorption % was found to be 0.76 and 1.18 for the ANFIS and ANN model respectively, which are much lower than the acceptable limit of 5%.
d) In terms of overall efficiency, the ANFIS model (R^2^ = 0.98) performed better than the ANN model (R^2^ = 0.93), though both models are satisfactory enough. This is maybe because of working with a small number of datasets. Working with an enormous number of datasets may exhibit more efficiency in the case of the ANN model.

The bright side of the research is that the models are customizable and capable of eliminating a lot of trial-and-error effort to predict the textile material's property. Furthermore, it can flourish the scalable production of functional textiles with minimum hassle and constriction regarding the desired property of the final product.

To develop and investigate the performance of the models in the future, i) more datasets, ii) other types of binders than acrylic, and iii) different types of fabrics can be considered.

## 5 Limitations

This paper only discusses the water absorption property of PU-treated polyester fabrics. But considering more properties would give a better insight into the behavior of the treated fabrics. Moreover, working with more data improves the prediction capability of the ANFIS and ANN models. The number of datasets presented in this paper is not far below of some other researchers, but working with more data would give more accurate results.

## Declarations

### Author contribution statement

Joy Sarkar: Conceived and designed the experiments; Analyzed and interpreted the data; Contributed reagents, materials, analysis tools or data; Wrote the paper.

Zawad Hasan Prottoy & Md. Tanimul Bari: Performed the experiments; Contributed reagents, materials, analysis tools or data.

Md Abdullah Al Faruque: Analyzed and interpreted the data; Wrote the paper.

### Funding statement

This research did not receive any specific grant from funding agencies in the public, commercial, or not-for-profit sectors.

### Data availability statement

Data will be made available on request.

### Declaration of interests statement

The authors declare no conflict of interest.

### Additional information

No additional information is available for this paper.
